# Association of Clinical, Biological, and Brain Magnetic Resonance Imaging Findings With Electroencephalographic Findings for Patients With COVID-19

**DOI:** 10.1001/jamanetworkopen.2021.1489

**Published:** 2021-03-15

**Authors:** Virginie Lambrecq, Aurélie Hanin, Esteban Munoz-Musat, Lydia Chougar, Salimata Gassama, Cécile Delorme, Louis Cousyn, Alaina Borden, Maria Damiano, Valerio Frazzini, Gilles Huberfeld, Frank Landgraf, Vi-Huong Nguyen-Michel, Phintip Pichit, Aude Sangare, Mario Chavez, Capucine Morélot-Panzini, Elise Morawiec, Mathieu Raux, Charles-Edouard Luyt, Pierre Rufat, Damien Galanaud, Jean-Christophe Corvol, Catherine Lubetzki, Benjamin Rohaut, Sophie Demeret, Nadya Pyatigorskaya, Lionel Naccache, Vincent Navarro

**Affiliations:** 1 Sorbonne Université, Paris Brain Institute, Institut du Cerveau, Institut National de la Santé et de la Recherche Médicale U 1127, Centre National de la Recherche Scientifique, Unité Mixte de Recherche 7225, Paris, France; 2 Assistance Publique des Hôpitaux de Paris, Clinical Neurophysiology Department, Pitié-Salpêtrière Hospital, Paris, France; 3 Neurophysiology Department, Sorbonne Université, Paris, France; 4 Neuroradiology Department, Sorbonne Université, Paris, France; 5 Assistance Publique des Hôpitaux de Paris, Neuroradiology Department, Pitié-Salpêtrière Hospital, Paris, France; 6 Assistance Publique des Hôpitaux de Paris, Neurology Department, Pitié-Salpêtrière Hospital, Paris, France; 7 Neurology Department, Sorbonne Université, Paris, France; 8 Service de Pneumologie, Sorbonne Université, Paris, France; 9 Assistance Publique des Hôpitaux de Paris, Service de Pneumologie, Médecine Intensive et Réanimation, Pitié-Salpêtrière Hospital, Paris, France; 10 Department of Anesthesia, Critical Care and Peri-Operative Medicine, Sorbonne Université, Paris, France; 11 Assistance Publique des Hôpitaux de Paris, Department of Anesthesia, Critical Care and Peri-Operative Medicine, Pitié-Salpêtrière Hospital, Paris, France; 12 Institut de Cardiologie, Sorbonne Université, Paris, France; 13 Sorbonne Université, Institut National de la Santé et de la Recherche Médicale, Unité Mixte de Recherche, 1166–Institute of Cardiometabolism and Nutrition, Paris, France; 14 Assistance Publique des Hôpitaux de Paris, Service de Médecine Intensive Réanimation, Institut de Cardiologie, Pitié-Salpêtrière Hospital, Paris, France; 15 Assistance Publique des Hôpitaux de Paris, Biostatistic Department, Pitié-Salpêtrière Hospital, Paris, France; 16 Center of Reference for Rare Epilepsies, Pitié-Salpêtrière Hospital, Paris, France

## Abstract

**Importance:**

There is evidence of central nervous system impairments associated with coronavirus disease 2019 (COVID-19) infection, including encephalopathy. Multimodal monitoring of patients with COVID-19 may delineate the specific features of COVID-19–related encephalopathy and guide clinical management.

**Objectives:**

To investigate clinical, biological, and brain magnetic resonance imaging (MRI) findings in association with electroencephalographic (EEG) features for patients with COVID-19, and to better refine the features of COVID-19–related encephalopathy.

**Design, Setting, and Participants:**

This retrospective cohort study conducted in Pitié-Salpêtrière Hospital, Paris, France, enrolled 78 hospitalized adults who received a diagnosis of severe acute respiratory syndrome coronavirus 2 (SARS-Cov2) and underwent EEG between March 30 and June 11, 2020.

**Exposures:**

Detection of SARS-CoV-2 from a nasopharyngeal specimen using a reverse transcription–polymerase chain reaction assay or, in the case of associated pneumonia, on a computed tomography scan of the chest.

**Main Outcomes and Measures:**

Data on the clinical and paraclinical features of the 78 patients with COVID-19 were retrieved from electronic patient records.

**Results:**

Of 644 patients who were hospitalized for COVID-19, 78 (57 men [73%]; mean [SD] age, 61 [12] years) underwent EEG. The main indications for EEG were delirium, seizure-like events, and delayed awakening in the intensive care unit after stopping treatment with sedatives. Sixty-nine patients showed pathologic EEG findings, including metabolic-toxic encephalopathy features, frontal abnormalities, periodic discharges, and epileptic activities. Of 57 patients who underwent brain MRI, 41 showed abnormalities, including perfusion abnormalities, acute ischemic lesions, multiple microhemorrhages, and white matter–enhancing lesions. Fifty-five patients showed biological abnormalities, including dysnatremia, kidney failure, and liver dysfunction, the same day as the EEG. The results of cerebrospinal fluid analysis were negative for SARS-Cov-2 for all tested patients. Nine patients who had no identifiable cause of brain injury outside COVID-19 were further isolated; their brain injury was defined as COVID-19–related encephalopathy. They represented 1% (9 of 644) of patients with COVID-19 requiring hospitalization. Six of these 9 patients had movement disorders, 7 had frontal syndrome, 4 had brainstem impairment, 4 had periodic EEG discharges, and 3 had MRI white matter–enhancing lesions.

**Conclusions and Relevance:**

The results from this cohort of patients hospitalized with COVID-19 suggest there are clinical, EEG, and MRI patterns that could delineate specific COVID-19–related encephalopathy and guide treatment strategy.

## Key Points

**Question:**

Can electroencephalography (EEG), combined with clinical, biological, and magnetic resonance imaging (MRI) analyses, help to better characterize patients with neurologic coronavirus disease 2019 (COVID-19) and diagnose specific COVID-19–related encephalopathy?

**Findings:**

Neurologic manifestations, biological findings, EEG findings, and brain MRI images were analyzed in a cohort study of 78 adult patients with COVID-19. Nine patients had no identified cause of brain injury, as revealed by biological and MRI findings; their injury was defined as COVID-19–related encephalopathy.

**Meaning:**

This study suggests that, although neurologic manifestations, EEG findings, and MRI findings may appear heterogeneous and nonspecific, multimodal monitoring may better identify patients with COVID-19–related encephalopathy and guide treatment strategy.

## Teaser

This cohort study investigates clinical, biological, and brain magnetic resonance imaging (MRI) findings in association with electroencephalographic (EEG) features for patients with coronavirus disease 2019 (COVID-19) and refines the features of COVID-19–related encephalopathy.

## Introduction

Severe acute respiratory syndrome coronavirus 2 (SARS-CoV-2) may damage the central nervous system (CNS).^1,2^ Brain magnetic resonance imaging (MRI) results or cerebrospinal fluid findings may be suggestive of encephalitis or may be normal for patients with CNS symptoms.^3,4^ Electroencephalography (EEG) is a tool to identify neurologic injury and understand underlying mechanisms. At the beginning of the coronavirus disease 2019 (COVID-19) pandemic, periodic EEG discharges with triphasic morphologic characteristics were reported in 1 patient with alteration of consciousness,^5^ with unremarkable results of cerebrospinal fluid analysis and brain MRI. Frontal periodic EEG discharges were further reported in 5 critically ill patients with COVID-19.^6^ To our knowledge, few studies have evaluated EEG findings together with clinical, biological, and MRI findings in patients with COVID-19, and these studies did not show evidence of specific patterns.^7,8,9^

Here, we aimed to better characterize patients with neurologic COVID-19 and, possibly, to identify a subgroup of patients with COVID-19–related encephalopathy (CORE). We combined EEG with clinical, biological, and MRI findings in a cohort study of 78 patients. We had 3 main goals: (1) to provide a description of the clinical symptoms and the biological, EEG, and MRI patterns observed in these patients, including their frequency and their prognostic value; (2) to analyze EEG patterns in light of MRI, clinical, and biological findings; and (3) to further define CORE.

## Methods

### Study Design and Participants

We included all consecutive adult inpatients with confirmed COVID-19^1^ (based on the results of a nasopharyngeal reverse transcription–polymerase chain reaction test or a chest computed tomography scan) who underwent EEG for neurologic symptoms in the Pitié-Salpêtrière Neurophysiology Department between March 30 and June 11, 2020. This study received approval from the Sorbonne Université Ethic Committee. All patients or relatives provided written consent. The study design and report are in accordance with the Strengthening the Reporting of Observational Studies in Epidemiology (STROBE) reporting guideline.

### Data Collection

Electroencephalography was performed over 20 minutes with SystemPlus Evolution (Micromed) using 8 to 21 channels, and the results were analyzed prospectively in a longitudinal bipolar montage.^10^ Demographic, clinical, and biological data were extracted from the electronic medical records. Clinical evaluation was performed before EEG, and then we reviewed neurologic symptoms and summarized into syndromes. For patients who had another neurologic evaluation before hospital discharge, we reported the proportion of patients with a total recovery of neurologic symptoms and the association of persistent neurologic symptoms with patient autonomy. Magnetic resonance imaging scans were performed using the 3.0-T MRI system (Premier; GE Healthcare) with a 48-channel receive head coil.^3^

Electroencephalographic features were analyzed in light of clinical and biological characteristics and the therapeutics received on the day of EEG. We performed a detailed analysis of biological findings and focused on all disturbances (hyponatremia, hypocalcemia, renal insufficiency, hepatic dysfunction, hypercapnia, and hyperosmolarity; Table 1^11^) that were able to induce an encephalopathy pattern during EEG. If one of these disturbances was reported, the patient was classified as a patient with biological abnormality. Similarly, we reported all drugs taken by the patient at time of EEG. If the patient was under sedation or taking drugs with possible CNS adverse effects (antibiotic, pain medication, or psychotropic medications), we considered the possibility that the EEG findings may be affected by these drugs.^12^

**Table 1.** Characteristics of Patients

Demographic and clinical findings	No./total No. (%)
Patients with positive RT-PCR results	64/72 (89)
Patients with SARS-CoV-2–associated pneumonia on CT scan results	59/68 (87)
COVID-19 severity, mean (SD)^a^	5.32 (1.30)
Deceased patients at discharge	7/78 (9)
Patients with medical comorbidities	63/77 (82)
Cardiovascular disease	40/77 (52)
Lung disease	9/77 (12)
Diabetes	25/76 (33)
Tobacco use	16/75 (21)
Obesity	16/76 (21)
Immunosuppression	2/75 (3)
Cancer	7/75 (9)
Patients requiring ICU admission	49/78 (63)
Delay between the first COVID-19 symptoms and ICU admission, mean (SD), d	12.4 (11.3)
Patients requiring antibiotics, neuroleptics, or anesthetics^b^	40/71 (56)
Cardiopulmonary arrest	4/78 (5)
Symptoms reported before EEG	
Anosmia	12/66 (18)
Agueusia	9/66 (14)
Headache	8/75 (11)
Delirium	44/78 (56)
Seizures	10/78 (13)
Dizziness	2/73 (3)
Visual disturbances	1/75 (1)
Oculomotor disorders	6/74 (8)
Movement disorders	15/72 (21)
Sleep disorders	4/72 (6)
Neurologic syndromes reported during hospital care	
Language disorder	16/78 (21)
Disorder of consciousness	28/78 (36)
Brainstem impairment	7/78 (9)
Cerebellar syndrome	5/78 (6)
Cognitive disorders	36/78 (46)
Frontal syndrome	15/78 (19)
Worsening of preexistent cognitive disorders	7/78 (9)
Psychiatric disorders	4/78 (5)
EEG findings	
Delay between the first COVID-19 symptoms and the EEG, mean (SD), d	29.4 (21.3)
EEG performed in ICU	41/78 (53)
EEG performed under anesthesia	23/71 (32)
EEG performed in intubated patients	14/78 (18)
Normal EEG	9/78 (12)
Abnormal background activity	63/78 (81)
Focal abnormality	35/78 (45)
Frontal	24/35 (69)
Temporal	11/35 (31)
Other	6/35 (17)
Epileptic activities (interictal, n = 4; seizures, n = 1)^c^	4/78 (5)
Periodic discharges	6/78 (8)
Encephalopathy pattern	23/78 (30)
MRI findings	
Patients who underwent MRI	57/78 (73)
Unremarkable MRI findings	16/57 (28)
Hemorrhages	21/57 (37)
Multiple microhemorrhages	10/21 (48)
Corpus callosum injury	4/21 (19)
Acute ischemic lesions	13/57 (23)
Gray matter injury	13/57 (23)
White matter enhancing lesions	5/57 (9)
Basal ganglia abnormalities	4/57 (7)
Hypoxic-ischemic lesions	3/57 (5)
Metabolic abnormalities	3/57 (5)
PRES lesions	2/57 (4)
Leptomeningeal contrast enhancement	2/57 (4)
CLOCC	1/57 (2)
Perfusion abnormalities	20/40 (50)
Hypoperfusion	19/20 (95)
Frontal hypoperfusion	14/19 (74)
Temporal hypoperfusion	10/19 (53)
Other location hypoperfusion	15/19 (79)
Hyperperfusion	4/20 (20)
Frontal hyperperfusion	3/4 (75)
Temporal hyperperfusion	4/4 (100)
Other location hyperperfusion	2/4 (50)
Biological findings the day of the EEG	
Patients with biological abnormalities^d^	55/77 (71)
Hyponatremia (sodium, <135 mEq/L)	11/76 (15)
Hypernatremia (sodium, >145 mEq/L)	13/76 (17)
Hypocalcemia (calcium, <8 mg/dL)	25/61 (41)
Renal insufficiency (according to creatinine clearance)	32/67 (48)
Hepatic dysfunction (AST and/or ALT 3 times higher than the standard)	10/65 (15)
Hypercapnia (>45 mm Hg)	13/45 (29)
Hyperosmolarity (>310 mOsm/kg)	13/40 (33)
Patients with lumbar puncture	30/78 (39)
Delay between the first COVID-19 symptoms and the lumbar puncture, mean (SD), d	29.2 (25.3)
Patients with positive RT-PCR results in CSF	0/26
Patients with increased CSF elements (>5/mm^3^)	3/30 (10)
Patients with increased CSF proteins (>0.65 g/L)	4/30 (13)

Abbreviations: ALT, alanine aminotransferase; AST, aspartate aminotransferase; CLOCC, cytotoxic lesion of the corpus callosum; COVID-19, coronavirus disease 2019; CSF, cerebrospinal fluid; CT, computed tomography; EEG, electroencephalogram; ICU, intensive care unit; MRI, magnetic resonance imaging; PRES, posterior reversible encephalopathy syndrome; RT-PCR, reverse transcription–polymerase chain reaction; SARS-CoV-2, severe acute respiratory syndrome coronavirus 2.

SI conversion factors: To convert sodium to millimoles per liter, multiply by 1.0; and calcium to millimoles per liter, multiply by 0.25.

^a^ COVID-19 severity was evaluated according to the World Health Organization nadir scale^11^: 1, nonhospitalized patients without activity limitation; 2, nonhospitalized patients with activity limitation; 3, hospitalized patients without oxygen requirement; 4, hospitalized patients with oxygen requirement; 5, hospitalized patients with noninvasive ventilation; 6, hospitalized patients with invasive ventilation; and 7, deceased patient at discharge.

^b^ We evaluated all drugs taken by patient the day of the EEG.

^c^ One patient had both interictal epileptic activities and seizures.

^d^ Patients were assessed as having biological abnormalities if they presented with one of the following abnormalities: hyponatremia, hypernatremia, hypocalcemia, renal insufficiency, hepatic dysfunction, hypercapnia, or hyperosmolarity.

### Statistical Analysis

Pairwise comparisons were performed using *t* tests or Mann-Whitney tests when appropriate to evaluate the association of an intensive care unit (ICU) stay with EEG findings or the prognostic significance of EEG alterations. We performed univariable logistic regression analyses to identify paraclinical variables that differ between patients with CORE and other patients. The sequential rejective Benjamini-Hochberg test procedure was used to correct for multiple comparisons. Next, in the cohort of the 57 patients who underwent both EEG and brain MRI, we performed a backward stepwise logistic regression procedure to select the variables most associated with CORE (n = 5).

We then used a multivariable logistic regression model with the 5 variables previously selected. The performance of the model was evaluated according to the area under the receiver operating characteristic curve. We also reported sensitivity and specificity. To evaluate the classification performance, we performed a 100-fold cross-validation. Our data set was partitioned into 2 folds: 70% of the patients were used for training, and 30% for testing. All *P* values were from 2-sided tests and results were deemed statistically significant at *P* < .05. Analyses were performed with R software, version 3.5.0 (R Foundation for Statistical Computing).

## Results

### Population Characteristics

During the inclusion period, 644 patients were hospitalized for COVID-19 in Pitié-Salpêtrière Hospital and 78 underwent EEG (57 men and 21 women; mean [SD] age, 61 [12] years; and mean [SD] delay after COVID-19 onset, 29 [21] days). Seven of the 78 patients (9%) died at hospital discharge. Forty-three of the 71 surviving patients (61%) underwent a new neurologic evaluation before hospital discharge; 15 patients (35%) had a total recovery of neurologic symptoms, while 28 patients (65%) had persistent neurologic symptoms. Among these patients, 22 were studied for autonomy at discharge, and 8 (36%) were considered fully dependent.

#### Clinical Findings

Before the patients underwent EEG, the most frequent neurologic manifestations were delirium (n = 44), movement disorders (n = 15, including tremor [n = 3], dyskinesia [n = 2], akathisia [n = 2], myorrhythmia [n = 2], and myoclonus [n = 8]), anosmia (n = 12), seizures (n = 10, including status epilepticus [n = 3], focal seizures [n = 2], and generalized seizures [n = 5]), and oculomotor disorders (n = 6) (Table 1^11^). At hospital discharge, all neurologic manifestations were summarized into syndromes, such as disorder of consciousness (n = 28), frontal syndrome (n = 15), brainstem impairment (n = 7), and cerebellar syndrome (n = 5).

#### EEG Findings

**Figure 1.**
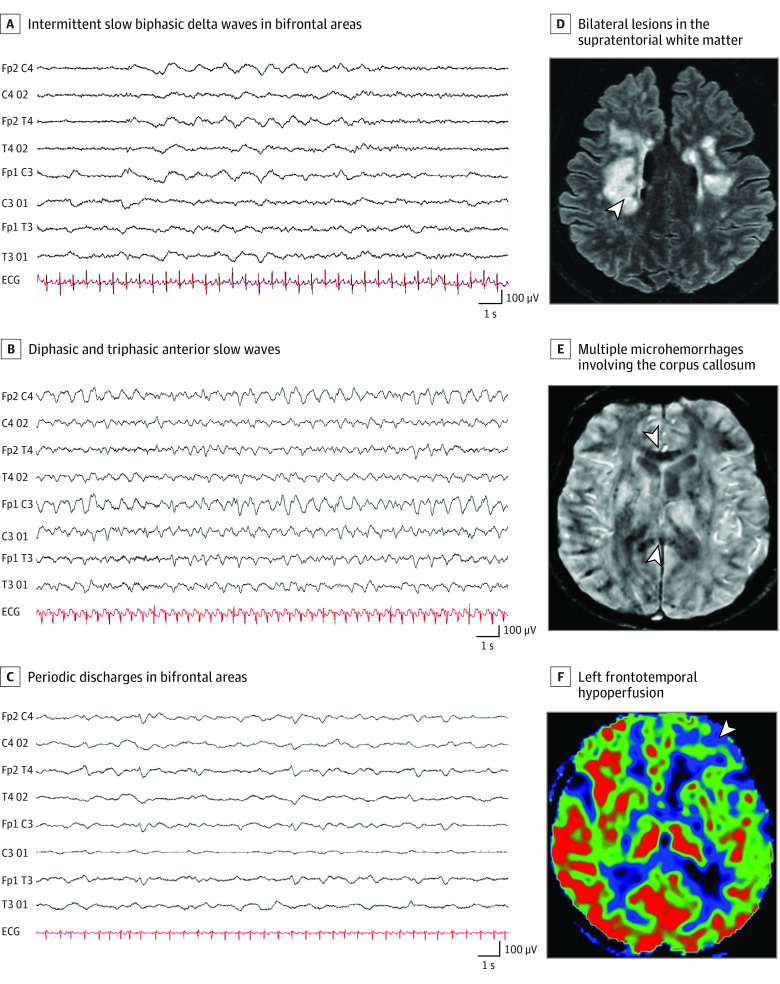
Examples of Electroencephalogram Recordings and Magnetic Resonance Imaging Findings Eight electrodes, longitudinal bipolar montage, 20-second epoch, low frequency filter 0.53 Hz, high frequency filter 70 Hz. A, Intermittent slow biphasic delta waves in bifrontal areas, with low-voltage continuous background activity. B, Diphasic and triphasic anterior slow waves, with slow continuous background activity, C, Periodic discharges in the bifrontal areas, with low voltage background activity, D, Bilateral lesions in the supratentorial white matter (arrowhead), hyperintense on axial fluid-attenuated inversion recovery images, E, Multiple microhemorrhages (arrowheads) involving the corpus callosum on T2 star images, F, Left frontotemporal hypoperfusion (arrowhead). ECG indicates electrocardiogram.

The main indications for EEG were delirium (24 of 78 [31%]), seizure-like events (22 of 78 [28%]), and delayed awakening after stopping sedatives (17 of 78 [22%]). Electroencephalographic abnormalities were identified in 69 patients: periodic discharges (n = 6); epileptic activities (n = 4); an abnormal EEG background without periodic EEG discharges, epileptic activities, or frontal slow waves (n = 12); and frontal slow waves (n = 47). The latter included an encephalopathy pattern (ie, reactive triphasic or rhythmic diffuse waves with bifrontal predominance; n = 23) and frontal slow waves (unilateral or bilateral and symmetric or nonsymmetric; n = 24) (Figure 1).

Patients in the ICU experienced more abnormal background activity than patients not in the ICU (39 of 41 [95%] vs 24 of 37 [65%]; *P* < .001). Nevertheless, the periodic discharges, epileptic activities, focal abnormalities, or encephalopathy patterns were seen in both ICU patients and non-ICU patients.

Among the 35 patients with focal abnormalities, 17 were studied for autonomy at discharge. Those with focal frontal abnormalities had a less-frequent total recovery of neurologic symptoms at hospital discharge than those with other abnormalities (1 of 10 [10%] vs 4 of 7 [57%]; *P* = .05). No EEG pattern was associated with death at hospital discharge.

#### MRI Findings

Of 57 patients who underwent MRI, 41 had abnormalities: acute ischemic lesions (n = 13), white matter–enhancing lesions (n = 5), basal ganglia abnormalities (n = 4), and metabolic lesions (ie, central pontine myelinolysis) (n = 3). Twenty patients had perfusion abnormalities—almost entirely hypoperfusion (n = 19) (Figure 1). The results of MRI scans were more frequently unremarkable than EEG findings (16 of 57 [28%] vs 9 of 78 [12%]; *P* = .02).

#### Drugs and Biological Findings

Electroencephalographic features were explained according to major confounders at the time of EEG. Fifty-five patients showed biological abnormalities, including dysnatremia, kidney failure, and liver dysfunction, the same day as the EEG procedure. Of 23 patients with encephalopathy, 7 received antibiotics, 1 received a neuroleptic drug, and 4 received light sedation on the day of EEG.^13^ Eighteen patients had biological abnormalities (moderate to severe renal insufficiency [n = 5], hypernatremia [n = 3], and hyponatremia [n = 1]).

No patients had positive reverse transcription–polymerase chain reaction results from cerebrospinal fluid samples (n = 26). No patients received immunomodulatory treatments before EEG.

### EEG Results and Related Clinical and Paraclinical Findings

The neurologic manifestations and MRI abnormalities were described according to EEG patterns (eTable in the Supplement). Owing to the large heterogeneity of clinical, MRI, and EEG findings, we were not able to show specific correlations between those findings. Three of 4 patients with epileptic activities detected by EEG had previous seizures, and 5 of 24 patients with EEG frontal abnormalities had frontal syndrome.

**Figure 2.**
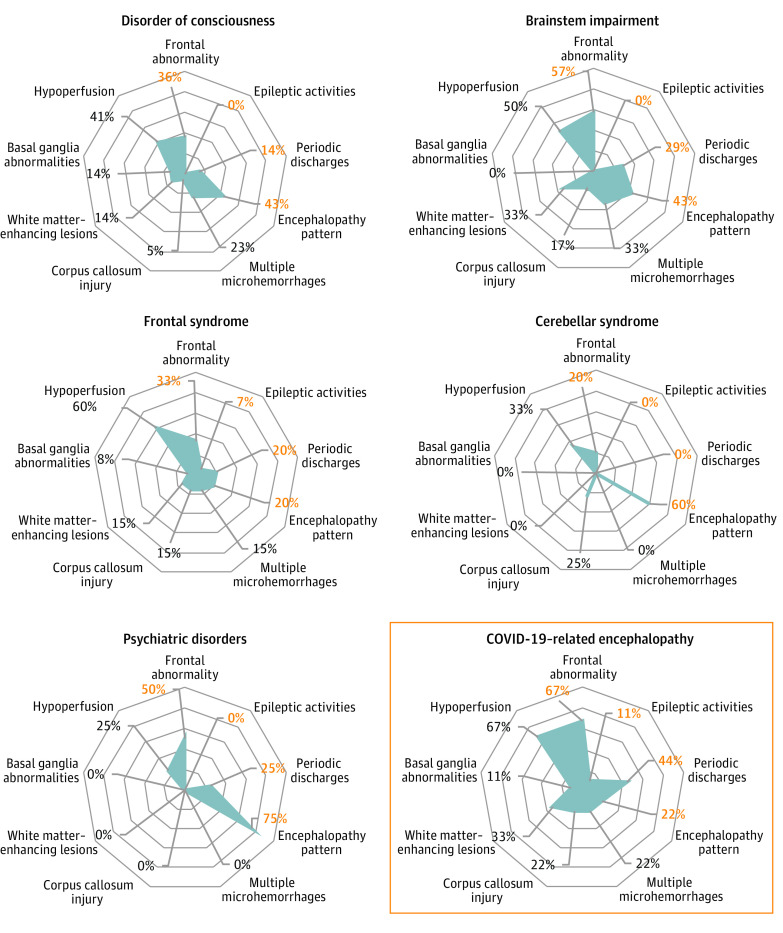
Representation of Electroencephalogram (EEG) and Brain Magnetic Resonance Imaging (MRI) Findings According to Neurologic Syndromes Each radar chart is a graphical representation of the proportion of patients with the selected neurologic syndrome who had EEG findings (frontal abnormality, epileptic activities, periodic discharges, and encephalopathy pattern; percentage in orange) or MRI findings (multiple microhemorrhages, corpus callosum injury, white matter–enhancing lesions, basal ganglia abnormalities, and hypoperfusion; percentage in black). Each concentric circle represents a proportion of 20%. A blue line is drawn connecting the values (percentages) for each finding (EEG or MRI), giving the blue polygon appearance. The polygon represents the prevalence of EEG and MRI findings: the larger the area of the polygon is, the more abnormal EEG or MRI findings the patients had. Data are represented as proportion to take into account missing data. COVID-19 indicates coronavirus disease 2019.

An overview of the most specific EEG and brain MRI findings is represented according to clinically defined syndromes in Figure 2. In our cohort, patients with disorder of consciousness, brainstem impairment, or frontal syndrome seemed to more frequently have EEG or MRI abnormalities than those with cerebellar syndrome or psychiatric disorders.

### Patients With COVID-19–Related Encephalopathy

To isolate a subgroup of patients with specific COVID-19–related brain injury, we distinguished patients with an identified cause of central neurologic disorders from those without. Based on clinical and paraclinical findings, the causes were as follows: ICU complications (n = 37), isolated metabolic or toxic encephalopathy (n = 8), cerebrovascular disorders (n = 6), previous mild cognitive impairment (n = 3), intracranial tumors (n = 2), isolated seizures and epilepsy (n = 6), history of psychiatric disorders (n = 3), cardiorespiratory arrest (n = 3), multiorgan failure (n = 2), associated varicella zoster virus encephalitis (n = 1), and headache (n = 1).

The 9 remaining patients who had acute neurologic injuries with duration more than 48 hours, without any identified cause of encephalopathy (clinical, MRI, or biological), were assessed as patients with CORE (Figure 2; Table 2). Compared with patients without CORE, those with CORE presented more frequently with movement disorders (6 of 9 [67%] vs 9 of 63 [14%]; *P* = .002), frontal syndrome (7 of 9 [78%] vs 8 of 69 [12%]; *P* < .001), brainstem impairment (4 of 9 [44%] vs 3 of 69 [4%]; *P* < .001), periodic EEG discharges (4 of 9 [44%] vs 2 of 69 [3%]; *P* < .001), and white matter–enhancing MRI lesions (3 of 9 [33%] vs 2 of 48 [4%]; *P* = .03).

**Table 2.** Characteristics of Patients With COVID-19–Related Encephalopathy

Characteristic	Patient No.									Unifying features
	1	2	3	4	5	6	7	8	9	
Age, y	69	66	52	50	60	49	72	56	61	NA
Sex	M	F	M	M	F	M	M	M	M	NA
COVID-19 severity	6	4	6	6	1	6	4	6	6	NA
ICU admission	1	0	1	1	0	1	0	1	1	6/9
Delirium	0	1	1	1	0	0	0	0	0	3/9
Seizures	1	0	0	0	0	0	0	0	0	1/9
Visual disturbances	0	0	0	0	0	0	0	0	0	0/9
Oculomotor disorders	1	0	0	0	0	1	1	1	1	5/9
Movement disorders	1	0	1	0	1	1	1	0	1	6/9
Language disorder	1	1	0	1	0	0	0	0	0	3/9
Disorder of consciousness	0	0	1	0	0	0	0	1	1	3/9
Brainstem impairment	0	0	1	0	0	1	0	1	1	4/9
Cerebellar syndrome	0	0	0	0	1	0	1	0	0	2/9
Cognitive disorders	1	1	1	1	1	1	1	0	1	8/9
Frontal syndrome	1	1	0	1	1	1	1	0	1	7/9
Psychiatric disorders	0	0	0	0	1	0	0	0	0	1/9
Treatment	IVIG	IVIG and CTC	NA	NA	CTC	PLEX and CTC	IVIG	CTC	PLEX and CTC	NA
CSF										
Elements	1	1	1	0	0	2	6	1	0	NA
Proteins	0.6	0.35	1.07	0.64	0.23	0.32	0.25	0.26	0.87	NA
Normal EEG results	0	0	0	0	1	0	0	0	0	1/9
Abnormal background rhythm	1	1	1	1	0	1	0	1	1	7/9
EEG focal impairment	1	1	0	0	0	1	1	1	1	6/9
Frontal	1	1	NA	NA	NA	1	1	1	1	6/6
Temporal	0	1	NA	NA	NA	0	0	0	0	1/6
Periodic discharges	1	1	0	0	0	0	0	1	1	4/9
Encephalopathy pattern	0	1	0	0	0	0	0	0	1	2/9
Epileptic activities	1	0	0	0	0	0	0	0	0	1/9
MRI performed	1	1	1	1	1	1	1	1	1	9/9
Hemorrhages	0	0	0	1	0	1	1	1	1	5/9
Microhemorrhages	NA	NA	NA	0	NA	1	0	1	0	2/5
Corpus callosum injury	NA	NA	NA	0	NA	1	1	0	0	2/5
Acute ischemic lesions	0	0	0	1	0	0	0	1	0	2/9
Gray matter injury	1	0	0	0	0	1	0	0	1	3/9
White matter–enhancing lesions	0	0	0	1	0	1	0	1	0	3/9
Basal ganglia abnormalities	1	0	0	0	0	0	0	0	0	1/9
Hypoxic ischemic lesions	0	0	0	0	0	0	0	0	0	0/9
Metabolic abnormalities	0	0	0	0	0	0	0	0	0	0/9
PRES lesions	0	0	0	0	0	0	0	1	0	1/9
Leptomeningeal contrast enhancement	0	0	0	0	0	0	0	0	0	0/9
CLOCC	0	0	0	0	0	0	0	0	0	0/9
Perfusion disorders	1	1	1	1	0	0	NA	NA	0	4/7
Hyperperfusion	0	0	0	0	NA	NA	NA	NA	NA	0/4
Hypoperfusion	1	1	1	1	NA	NA	NA	NA	NA	4/4
Frontal	1	1	1	1	NA	NA	NA	NA	NA	4/4
Temporal	0	0	0	1	NA	NA	NA	NA	NA	1/4

Abbreviations: CLOCC, cytotoxic lesions of the corpus callosum; COVID-19, coronavirus disease 2019; CSF, cerebrospinal fluid; CTC, corticosteroids; EEG, electroencephalogram; ICU, intensive care unit; IVIG, intravenous immunoglobulins; MRI, magnetic resonance imaging; NA, not applicable; PLEX, plasma exchange; PRES, posterior reversible encephalopathy syndrome.

**Figure 3.**
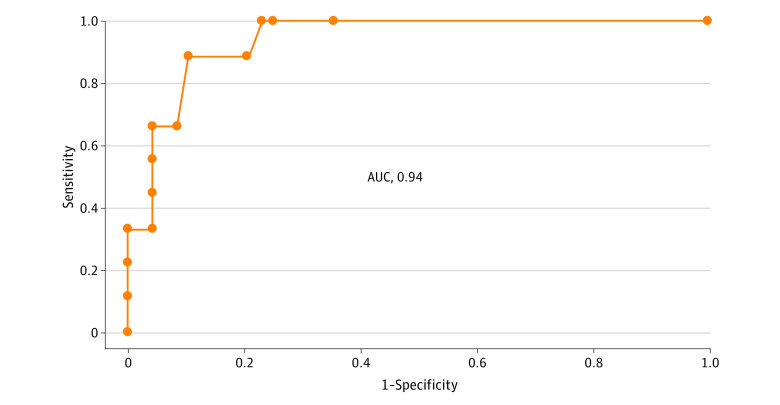
Clinical, Electroencephalogram (EEG), and Magnetic Resonance Imaging (MRI) Disturbances in Patients With Coronavirus Disease 2019 (COVID-19)–Related Encephalopathy Receiver operating characteristic curve for the model, evaluating the performance of movement disorders, brainstem impairment, frontal syndrome, EEG periodic discharges, and white matter–enhancing MRI lesions to identify patients with COVID-19–related encephalopathy. AUC indicates area under the curve.

Using clinical, EEG, and MRI data, we developed a model to identify patients with CORE, taking into account variable risk. The regression model included periodic EEG discharges, movement disorders, brainstem impairment, frontal syndrome, and white matter–enhancing MRI lesions. The model resulted in an area under the receiver operating characteristics curve of 0.94 (95% CI, 0.88-1.00; *P* < .001) (Figure 3). After a 100-fold cross-validation, the model was able to estimate the risk for a new patient to present with CORE with a sensitivity of 76% (95% CI, 33%-100%), specificity of 93% (95% CI, 86%-100%), positive predictive value of 65% (95% CI, 33%-100%), negative predictive value of 95% (95% CI, 86%-100%), and accuracy of 91% (95% CI, 76%-100%).

## Discussion

We report on a cohort of 78 patients with COVID-19 who underwent EEG for a wide range of CNS manifestations. Recent case reports, small series, and meta-analyses assessing the value of EEG for patients with COVID-19^5,6,7,8,9,14,15,16,17^ showed (1) nonspecific patterns, reflecting the diversity of SARS-CoV-2 infection complications,^1,7,9,14,15,16,17^ or (2) a striking periodic EEG pattern,^5,6,8,17^ suggestive of COVID-19–specific brain complications. Nevertheless, a systematic correlation of EEG findings with biological findings and brain MRI findings was lacking, precluding a better understanding of pattern origins.

We performed a multimodal evaluation of patients with COVID-19. Their neurologic complications were sometimes associated with ICU complications, preexisting pathologic conditions, toxic or metabolic encephalopathies, or strokes.^1,14^ The existence of specific COVID-19 brain complications is still being debated. Eight patients with SARS-CoV-2 infection and irritability, delirium, drowsiness, and new-onset epilepsy were reported.^14^ Additional reports further reinforced the hypothesis of brain-specific COVID-19 involvement, including marked brain metabolism changes detected on fluorodeoxyglucose positron emission tomography scans.^18^ We defined this brain involvement as CORE. In our study, we showed that patients with CORE mostly had movement disorders (mainly seizures and/or myorrhythmia), and brainstem impairment (oculomotor disorders such as bobbing) and frontal syndrome (disinhibition and grasping).

Similarly, MRI findings showed both (1) unspecific lesions, such as perfusion abnormalities, and (2) more specific lesions, such as basal ganglia abnormalities, microhemorrhages, corpus callosum injury, and white matter–enhancing lesions.^3^ The latter abnormality was the most significant lesion detected on MRI scans in patients with CORE (Table 2).

The most frequent EEG findings were abnormal background activity (81%) and frontal slow waves (60%). The latter were associated with metabolic and toxic encephalopathies—for which we identified 1 or several factors in most cases—or frontal lesions. Six patients (8%) showed a periodic EEG pattern, predominating in frontal lobes and not explained by MRI findings.

Our results are in accordance with previous reports; a recent meta-analysis reported abnormal background activity in almost all patients (96.1%),^16^ while half of all patients had focal slowing that involved the frontal region.^17^ A more specific periodic EEG pattern was also reported, with an incidence ranging from 0% to 38% according to the etiologic characteristics.^6,7,8,9,15,16,19,20,21,22,23^ Nevertheless, we found that this periodic EEG pattern had no prognostic value.

Epileptiform discharges and seizures have been reported in patients with COVID-19, with an incidence ranging from 0% to 63% for epileptiform discharges and from 0% to 25% for seizures.^7,8,9,15,16,19,20,21,22,23,24,25^ In our cohort, 4 patients (5%) had epileptiform discharges, and seizures occurred in 1 patient (1%) during EEG.

Patients with CORE had a periodic EEG pattern more frequently than other patients. All EEG abnormalities from the frontal lobe, coupled with the frontal syndrome noted in patients with CORE, suggest frontal lobe dysfunction, which is reminiscent of the hypothesis of a neuroinvasive entry of SARS-CoV-2 into the brain via the olfactory nerves or via the nasopharyngeal mucosa.^18,26^ A change in neuronal excitability, perhaps mediated by specific cytokines, may occur in brain areas close to the nasopharynx, such as the orbitofrontal lobe and the brainstem. Because inflammatory mechanisms, such as cytokine-mediated response or postviral autoimmune process, are suspected, immunomodulator treatments, such as plasma exchanges or intravenous immunoglobulins, may be proposed as early treatment for patients with CORE (Table 2).^26,27,28^

### Limitations

This study has some limitations. A relatively small number of patients underwent both EEG and MRI in a single center. There was a lack of systematic follow-up after hospital discharge. There was also a risk of underestimating the number of patients with CORE owing to other COVID-19–related comorbidities or pathologic conditions.

## Conclusions

Despite different clinical presentations, our study suggests that EEG is a valuable procedure for patients with COVID-19 and neurologic symptoms, to better identify different brain dysfunctions and CORE. We further emphasize the benefit associated with combining EEG and brain MRI for patients with neurologic symptoms concomitant with COVID-19. It remains to be clarified whether treatment strategies could be optimized with earlier identification of patients with CORE.

## Notes

Supplement. **eTable.** EEG Results and Related Clinical and Paraclinical Findings
