# Group Members Added to Supplement

**DOI:** 10.1001/jamanetworkopen.2022.19899

**Published:** 2022-06-16

The Original Investigation titled “Association of Clinical, Biological, and Brain Magnetic Resonance Imaging Findings With Electroencephalographic Findings for Patients With COVID-19,”^1^ published March 15, 2021, has been corrected to include the nonauthor collaborator names in a supplement.
