# Supplementary material for: Association of Clinical, Biological, and Brain Magnetic Resonance Imaging Findings With Electroencephalographic Findings for Patients With COVID-19
Source: JAMA Netw Open. 2021 Mar 15;4(3):e211489. doi: 10.1001/jamanetworkopen.2021.1489 (PMC7961310; doi:10.1001/jamanetworkopen.2021.1489)
Supplement: Supplement. — eTable. EEG Results and Related Clinical and Paraclinical Findings [file jamanetwopen-e211489-s001.pdf]

Supplementary Online Content

Lambrecq V, Hanin A, Munoz-Musat E, et al; Cohort COVID-19 Neurosciences (CoCo Neurosciences) Study Group. Association of clinical, biological, and brain magnetic resonance imaging findings with electroencephalographic findings for patients with COVID-19. *JAMA Netw Open.* 2021;4(3):e211489. doi:10.1001/jamanetworkopen.2021.1489

eTable. EEG Results and Related Clinical and Paraclinical Findings

This supplementary material has been provided by the authors to give readers additional information about their work.

© 2021 Lambrecq V et al. *JAMA Network Open.*

eTable. EEG Results and Related Clinical and Paraclinical Findings

**EEG**<br>78 patients / 57 patients with MRI<br>**Clinical and MRI findings**	Unremarkable EEG	Abnormal background activity	Focal abnormality				Epileptic activities (interictal, seizures)	Periodic discharges	Encephalopathy pattern
			All	Frontal	Temporal	Other			
**All patients (n=78)**	**9**	**63**	**35**	**24**	**11**	**6**	**4**	**6**	**23**
Delirium (n=44)	3	37	19	13	8	3	2	2	11
Seizures (n=10)	1	9	5	3	1	1	3	1	4
Movement disorders (n=15)	3	11	7	7	0	0	1	2	4
Language disorder (n=16)	1	13	8	5	3	2	1	2	6
Disorder of consciousness (n=28)	1	27	13	10	3	2	0	4	12
Brainstem impairment (n=7)	0	7	4	4	0	1	0	2	3
Cerebellar syndrome (n=5)	1	3	1	1	0	0	0	0	3
Cognitive disorders (n=36)	4	27	21	14	6	4	2	4	11
Frontal syndrome (n=15)	3	9	9	5	3	2	1	3	3
Psychiatric disorders (n=4)	1	4	3	2	1	1	0	1	3
**Patients with MRI (n=57)**	**7**	**45**	**29**	**20**	**8**	**5**	**2**	**6**	**17**
Unremarkable MRI (n=16)	3	13	8	6	2	1	0	1	5
Hemorrhages (n=21)	3	15	13	9	3	2	0	3	3
Multiple microhemorrhages (n=10)	1	8	8	6	3	2	0	2	1
Corpus callosum injury (n=4)	0	3	4	3	1	0	0	1	1
Acute ischemic lesions (n=13)	2	9	6	3	2	2	1	2	4
Gray matter injury (n=13)	2	10	7	5	1	1	2	2	4

© 2021 Lambrecq V et al. *JAMA Network Open.*

White matter enhancing lesions (n=5)	0	5	4	3	1	1	0	1	0
Basal ganglia abnormalities (n=4)	0	4	1	1	0	0	1	1	1
Hypoxic-ischemic lesions (n=3)	1	2	0	0	0	0	0	0	1
Metabolic abnormalities (n=3)	0	3	1	1	0	0	0	0	3
PRES lesions (n=2)	0	2	1	1	0	1	0	1	1
Leptomeningeal contrast enhancement (n=2)	0	2	0	0	0	0	0	0	0
Cytotoxic lesion of the corpus callosum (CLOCC) (n=1)	0	1	0	0	0	0	0	0	0
Perfusion abnormalities (n=20)	2	17	6	4	2	1	1	2	8
Hypoperfusion (n=19)	2	16	6	4	2	1	1	2	7
Hyperperfusion (n=4)	1	3	0	0	0	0	0	0	2

Data are represented as absolute numbers (the number of patients with each EEG findings and clinical or MRI findings).

© 2021 Lambrecq V et al. *JAMA Network Open.*
